# Impact of peak expiratory flow rate in general and regional anesthesia: A comparative study

**DOI:** 10.6026/973206300211082

**Published:** 2025-05-31

**Authors:** Dinesh M., Suganya S., Yugesh Kesavamoorthy, Sowmiya T., Marimuthu M., Sangeetha S.

**Affiliations:** 1 Department of Respiratory Care Technology, Dr. MGR Educational and Research Institute, Velapanchavadi, Chennai- 95, India; 2 Department of Physiology, Meenakshi Ammal Dental College, Alapakkam, Maduravoiyal, India; 3 Department of Anatomy, Sri Ramachandra Institute of Higher Education & Research (DU), Porur, Chennai - 600 116, Tamilnadu, India; 4 Department of biochemistry, Indira medical College and hospital, Thiruvallur, Tamilnadu, India; 5 Department of Anesthesia, Saveetha Medical College, Thandalam, Tamilnadu, India

**Keywords:** Peak expiratory flow rate (PEFR), general anesthesia, regional anesthesia

## Abstract

Anesthesia can have a significant impact on Peak expiratory flow rate (PEFR) due to several factors that leads to partial airway blockage and restriction air flow making it harder to exhale forcefully. A sample size of 150 study participants was taken, among which 75 subjects were undergone general anesthesia and 75 subjects undergone regional anesthesia. We categorize the participants into 2 groups, where group 1 receives general anesthesia and group 2 receives regional anesthesia. The subjects were selected based on inclusion and exclusion criteria. PEFR (336.02 ± 52.35) and Respiratory rate (16.07 ± 2.42) were found to be drastically reduced in post-operative when compared to pre-operative which was statistically significant (P<0.05). Therefore, it is observed that PEFR is significantly reduced in patients receiving general anesthesia during post-operative period where there is no much change in regional anesthesia.

## Background:

Respiratory system is the vital organ system which is required for ventilation and gas exchange in the alveoli. Effective ventilation is influenced by various factors such as respiratory center, the neuromuscular junction, respiratory muscle power, respiratory rate and tidal volume, respiratory compliance and resistance, bronchial muscle tone, ciliary movement and adequate reflexes [1]. A series of factors such as smoking, alcohol, pollution, allergens, genetic nutrition and stress which may impair the lung function [2]. Anesthetic drugs used for induction has an ability to interfere with oxygen intake. Induction of anesthesia and position during surgery affects the normal respiratory function [3]. Both general and regional anesthesia has an impact on ventilation. Volatile agents and intravenous anesthesia used in general anesthesia causes depression of respiratory centers in pons and medulla [4]. Administration of narcotics and inhalational agents causes hyperbaric due to shift of carbon di oxide curve to the right. It also causes ventilation perfusion mismatch which might leads to hypoxia and post-operative pulmonary complications [5]. General anesthesia could cause atelectasis due to decreased ventilation, high concentration of oxygen during pre-oxygenation and apnea. However insufflation of CO2 into abdomen causes peritoneal acidosis and hypercarbia during general anesthesia. Regional anesthesia can be administered to the patient with impaired lung function, posted for elective surgery. Administration of isoflurane causes decrease in PaO2 and increases the PaCO2, when compared to propofol [6]. The administration of general anesthesia reduces respiratory muscle tone, which may lead to airway obstruction. General anesthetic abolishes the respiratory reflex which might lead to laryngospasms and bronchospasm. It also inhibits the ciliary movement in the respiratory system. Spinal anesthesia is a common technique employed in lower abdomen surgery which recovers the lung function rapidly when compared to General anesthesia [7]. In healthy individuals, central neuraxial blockade does not affect pulmonary function. However high spinal anesthesia could leads to paralysis of the abdominal muscles reduces expiratory reserve volume and potentially impairs the coughing reflex [8]. A spinal block above T6 significantly decreases the FEV1, forced vital capacity (FVC) and forced expiratory flow (25-75%) in elderly patients with limited respiratory reserve [9]. Tidal volume (TV) generally remains unaffected unless the phrenic nerve is involved. In patients with severe respiratory disease, inspiratory muscles can able to sustain ventilation, but paralysis of expiratory muscles may hinder the effective coughing and clearance of pulmonary secretions [10].

Tidal volume, Minute volume and arterial oxygen tension were remains unaffected in regional anesthesia but high spinal could cause apnoea and respiratory depression. Incidence of Respiratory complications like bronchospasm, laryngospasm, post-operative atelectasis and hypoxia are very minimal in regional anesthesia when compared to general anesthesia [11]. Hence anesthesia has a profound effect on lung function, it is mandatory to assess lung function pre and post operatively. Lung function can be evaluated by measuring Peak expiratory flow rate (PEFR) is an authentic indicator of ventilation as well as airflow obstruction. Peak expiratory flow (PEF) is a volume of air expelled forcefully from the lungs in liters per minute which ranges from approximately 400 to 700 liters per minute [12]. PEFR is depends on various factors such as gender, thoracic dimensions (both intra- and extrathoracic), alveolar expansion capacity, lung elastic recoil, voluntary effort and respiratory muscle strength [12]. Hence comparing preoperative and postoperative PEFR values can helps to assess the impact of general anesthesia on lung function and respiration. Peak expiratory flow rates have been utilized to assess cough effectiveness in general surgical patients receiving extradural anesthesia [13]. Therefore, it is of interest to show the effect of PEFR during pre and post operatively in patients undergoing general and regional anesthesia.

## Materials and Methods:

The prospective clinical study was performed in 150 subjects among both males and females in the age group of 25-50 years. This study was conducted in Department of Anesthesiology, A.C.S Medical College & Hospital and Sri Lalithambigai Medical College and Hospital after obtaining Institutional Ethical Committee Approval. The study participants attending Operation theatre for elective surgery were explained about the procedure and benefits of this study. Informed consent was obtained from each volunteer. The detailed medical history of the participants were collected using questionnaire that includes name, age, occupation, H/O of co-existing disease and personal habits like H/O of smoking and alcohol. The subjects were selected based on inclusion and exclusion criteria. PEFR were recorded using Wrights peak flow meter in sitting position. The subject was instructed to take deep inspiration and asked to blow out forcefully through the mouth piece. Test manoeuvre was repeated thrice and the best result was considered for analysis. The Datas are expressed as mean ± S.D. A Student T test was conducted to compare and see the effect of PEFR in patients receiving general and regional anesthesia.

## Inclusion criteria:

[1] Both males and females undergoing elective surgeries

[2] Age group 25 years to 50 years

[3] Patients undergoes surgery under general anaesthesia

[4] Patients undergoes surgery under regional anaesthesia

## Exclusion criteria:

[1] Patient refusal

[2] Uncooperative patient

[3] Patient with H/O cardiac disease/ lung disease/Diabetes mellitus/ thyroid disorders

[4] BMI >30

[5] ASA grade III and IV

[6] Patients on steroid therapy

## Results:

The data were expressed as mean ± SD. Student T test were performed for all variables. Datas were analysed using SPSS version 19. Gender distribution of study participants were given in Table 1 and Figure 1 (see PDF) depicts the same. Distribution of male and female participants was more or less equal in group I whereas group 2 shows higher percent of female population than male. Age, height and weight were found to be similar among group 1 and group 2 which was depicted in Table 2. Variables such as PEFR, SBP, DBP, SPO2, PR and RR were shown in Table 3 along with graphical representation (Figure 2 - see PDF & Figure 3 - see PDF). PEFR were found to be drastically reduced in post-operative (336.02±52.35) when compared to pre-operative (411.12±52.05) which was statistically highly significant (P<0.001). But there was no much statistical significance among group 1 and group 2 which was shown in graph 2. Respiratory rate was observed to be reduced in post-operative period (16.07±2.42) when compared to pre-operative period (18.76±3.11) which was statistically significant (P< 0.05). There were no much significant changes between group 1 and group 2. Other variables such as SPO2, SBP, DBP and PR were found to be statistically insignificant between pre and post-operative among group 1 and group 2 which was shown in Figure 3 & 4 (see PDF).

## Discussion:

Peak expiratory flow rate (PEFR) is a simple and widely used method to evaluate lung function. It measures the speed of air expelled from the lungs which reflects the airway caliber and provide us a valuable tool for diagnosis of lung functions. Several factors influence PEFR that includes airway resistance, maximal voluntary effort and gender. Some of the research work analyzed the interaction between anesthetic drugs and lung function. Anesthetic drugs employed in general and regional anesthesia has adverse effect on lung function. PEFR were found to be decreased on administration of midazolam and propofol and the result was statistically significant (P<0.001) where the similar result were observed in this study (P<0.001) [14]. According to Montravers *et al.* investigated the consequences of intravenous drugs on upper airway resistance. They estimated the supraglottic pressures using a balloon-tipped catheter and airflow with a pneumotachograph. Although their approach for assessing respiratory function differed from ours, their conclusion was consistent with our findings. They suggested that the increased upper airway resistance following induction was due to reduced pharyngeal muscle tone [15]. The alterations in pulmonary function caused by spinal anesthesia were more significant than those seen after premedication, especially in MEF25-75 and PEFR values. Since these values are not influenced by patient cooperation, the consistent result was observed in our study were likely due to regional anesthesia (371.09 ± 37.25) rather than any decline in patient performance resulting from the sedative effects of premedication. Forced expiratory flow (25-75%) was found to be significantly decreased in geriatric patients and patients with poor respiratory reserve in high spinal anaesthesia. Al-Kaisy and colleagues documented a reduction in respiratory dysfunction in a volunteer population receiving 10 ml of 0.5% or 0.25% bupivacaine which correlates with our study [16]. Spinal anaesthesia (SA) provides fast and reliable segmental anaesthesia with minimal risk for respiratory depression [17]. A combination of sedation and high block predispose to a higher degree of desaturation. To overcome this profound effect on PEFR, we can avoid sedative premedication and block height could be maintained at T10 level by administrating low dose of local anaesthetic in both groups [18]. Spinal anaesthesia had an even greater impact on PEFR, showing a reduction of more than 35% in obese class I and II patients and over 45% in obese class III patients [19]. Our study differed from these findings because obese patients were excluded, resulting in preserved PEFR among our participants. Hence in this present study, we enlighten the effect of PEFR in general and regional anaesthesia. PEFR were found to be drastically reduced in post-operatively among group 1 when compared to group 2 (P<0.01). Vitals such as BP, SPO2 and PEFR were found to remains unaffected in patients receiving regional anaesthesia and reduced in general anaesthesia which was statistically significant (P<0.05).

## Conclusion:

Drugs used in general anesthesia have high impact on respiratory depression which could lead to decrease in PEFR. Regional anesthesia has minimal effect on lung function but level of blockade above T10 could lead to respiratory depression due to blockade of phrenic nerve. Hence assessment of lung function preoperatively is mandatory for patient attending operation theatre for surgery. Since anesthetic technique (General anesthesia/Regional anesthesia) has great impact on lung function this alters the expiratory flow rate. This enables the anesthetist to assess the efficiency of patient's lung before administrating the anesthetic drugs which is essential for safe anesthesia.

## Figures and Tables

**Table 1:** Gender distribution of study participants

**Number of Patients**	**Group**		**Total**
	**Group 1 (GA)**	**Group 2 (RA)**	
Male	28 (37.3%)	3(4%)	31(20.7%)
Female	47(62.7%)	72(96%)	119(79.3%)
Total	75(100%)	75(100%)	150(100%)

**Table 2:** Demography

	**Group 1 (GA)**		**Group 2 (RA)**		**p Value**
	**Mean**	**SD**	**Mean**	**SD**	
Age	35.61	7.89	31.84	6.58	0.001
Height	161.16	9.01	156.96	7	0.001
weight	57.86	3.1	59.33	3.3	0.001
Values indicate Mean ± SD
Data expressed in mean and SD using Student t test

**Table 3:** PEFR and other vitals among Group I and Group II during pre op and post op

**Parameters**	**Period**	**Group 1 (GA)**		**Group 2 (RA)**		**P Value**
	**Mean**	**SD**	**Mean**	**SD**	
RR	Pre-operative	18.76	3.11	18.15	2.54	0.08
	Post-operative	16.07	2.42	16.97	2.33	0.01*
SPO2	Pre-operative	98.81	1.24	98.98	0.92	0.17
	Post-operative	97.62	1.35	98.33	1.39	0.01*
PEFR	Pre-operative	411.12	52.05	394.12	37.07	0.01*
	Post-operative	336.02	52.35	371.09	37.25	0.001***
Systole	Pre-operative	124.44	12.79	122.75	9.59	0.18
	Post-operative	118.77	9.72	118.04	6.34	0.292
Diastole	Pre-operative	79.17	7.61	80.06	6.59	0.22
	Post-operative	75.76	8.03	78.42	4.35	0.01**
PR	Pre-operative	85.29	11.43	83.58	8.46	0.15
	Post-operative	77.39	12.6	76.02	8.79	0.2
Values indicate Mean ± SD,
*P<0.05, **P<0.01, ***P<0.001
